# A blue blood toddler– a case report of methemoglobinemia and literature review

**DOI:** 10.1186/s13052-025-01886-z

**Published:** 2025-02-21

**Authors:** Dorina Pjetraj, Madiha El Mechri, Silvia Bacelli, Elisabetta Fabiani, Laura Caponi, Simona Gatti, Elena Lionetti

**Affiliations:** 1 Department of Pediatrics, Marche Polytechnic University, Ancona, 60123 Italy; 2 Department of Pediatric Emergency, Salesi Hospital, Azienda Ospedaliero-Universitaria delle Marche, Ancona, Italy

**Keywords:** Methemoglobinemia (MetHb), Poisoning, Methylene blue, Ascorbic acid, Food-induced methemoglobinemia, Case report

## Abstract

**Background:**

Methemoglobinemia (MetHb) is a rare and potentially life-threatening condition caused by oxidation of ferrous hemoglobin (Fe2+) to the ferric (Fe3+) state, making it incapable of binding oxygen and resulting in cyanosis and tissue ischemia.

**Case presentation:**

This case presentation describes a 1-year-old boy who developed sudden cyanosis and reduced consciousness disorder. An initial assessment showed decreased oxygen saturation (SpO2 85%) despite oxygen therapy, while point-of-care venous blood gas (VBG) analysis assessed high rates of MetHb (72.7%). Methylene blue and ascorbic acid were administered, resulting in in rapid clinical recovery and normalized VBG test results. The trigger for this condition was not identified, however the most likely cause of poisoning was attributed to food oxidants.

**Conclusion:**

Starting from the description of a clinical case, this paper discusses the causes and mechanisms of possible poisoning and reviews recent guidelines for methemoglobinemia management.

## Background

Methemoglobinemia (MetHb) is a rare and potentially fatal condition caused by the oxidation of ferrous hemoglobin (Fe^2+^) to the ferric (Fe^3+^) state. Acquired MetHb may cause cyanosis and tissue ischemia unresponsive to oxygen supplementation [1]. This case report describes the diagnosis and management of a one-year-old boy presenting with methemoglobinemia in our pediatric emergency department and emphasizes the importance of early recognition and treatment of MetHb through a detailed review of the most recent scientific literature.

## Case presentation

A one-year-old boy of Tunisian descent was brought to the Pediatric Emergency Department, presenting with cyanosis, drowsiness, and desaturation. During the initial assessment, the patient was alert and responsive, with patent airways and a normal breathing pattern. Cardio-thoracic and abdominal examinations were unremarkable. The patient was found to have a blood pressure within the normal range (98/50 mmHg), mild tachycardia (HR: 165/min), tachypnea (RR: 50 breaths per minute) and hypoxia (SpO_2_ 85%), despite administration of 100% oxygen via facemask. His past medical history revealed prematurity (born at 32 gestational weeks) with normal growth and neurological development. There was no parental consanguinity. He had a flat angioma on the left hemisphere, which was under follow-up. The mother reported that before the symptoms started, the child had been playing at home in a well-ventilated room. He had no known allergies and was not taking any medications. There were no recent symptoms such as cough, fever, or other concerns. His last meal, consumed about one hour before the event, consisted of beef meat and Swiss chard. No other person in the family had consumed the same foods.

Upon obtaining an arterial blood sample, the color of the blood was noted to be dark brown. Venous blood gas analysis demonstrated abnormal findings including markedly decreased PO_2_ (9 mmHg), reduced oxygen saturation (SO_2_ 23.9%), elevated lactate levels (4.5 mmol/L), and a significantly elevated methemoglobin (MetHb) concentration (72.7%). pH was 7.33 and PCO_2_ 43 mmHg. The patient’s hemoglobin level was 11.8 g/dL, and the glucose level was 121 mg/dL. Chest X-ray was unremarkable. Approximately 15 min after the patient’s arrival, his clinical condition rapidly deteriorated. He became drowsy and experienced seizures, with oxygen saturation dropping as low as 70%.

At this point the patient received intravenous methylene blue (MB) at a dose of 2 mg/kg over 5 min, which was repeated after 15 min. This intervention led to a rapid improvement in the patient’s clinical status, including normalization of consciousness, skin color, and oxygen saturation levels. The patient was then transferred to the Pediatric Intensive Care Unit for close monitoring. A blood gas analysis performed 3 h later demonstrated a significant reduction in MetHb levels to 2.9%. Throughout the observation period, the child’s overall condition remained consistently good. Treatment was continued with the administration of ascorbic acid (500 mg given twice daily for a total of 16 doses). The following day, MetHb levels had returned to the normal range at 1% and remained stable during continued observation. Subsequent tests indicated normal renal and hepatic parameters [S-urea 25 (20–45) mg/dl, S-creatinine 0.34 (0.20–1.3) mg/dl, bilirubin total 0.2 (0.2–1.2) mg/dL and direct 0.1 (0–0.4) mg/dL; alanine transaminase 34 (5–40) units/L; aspartate transaminase 55 (5–40) units/L]. Inflammatory markers, such as C-reactive protein and procalcitonin, were negative. The microbiological examinations of stool specimens (stool culture, Enterobacteria, Enterovirus, Adenovirus, Rotavirus) resulted in negative outcomes. Additionally, the patient’s glucose-6-phosphate dehydrogenase (G6PDH) activity was within the normal reference range (12.9 (> 9.4) U/gr Hb) and analysis of hemoglobin variants with HPLC method did not reveal any abnormalities.

Given the normal basal levels of MetHb, clinicians decided not to pursue further genetic investigations and instead focused on acquired causes of MetHb. Since the child had not taken any medications or been exposed to any new substances, there was suspicion of food poisoning. The case was reported to public health authorities, who conducted a thorough analysis on the meat that the child had for lunch; however, they found no evidence of contamination. Unfortunately, it was not possible to investigate the vegetables as they had all been consumed.

## Discussion and conclusions

### Pathophysiology

**Fig. 1:**
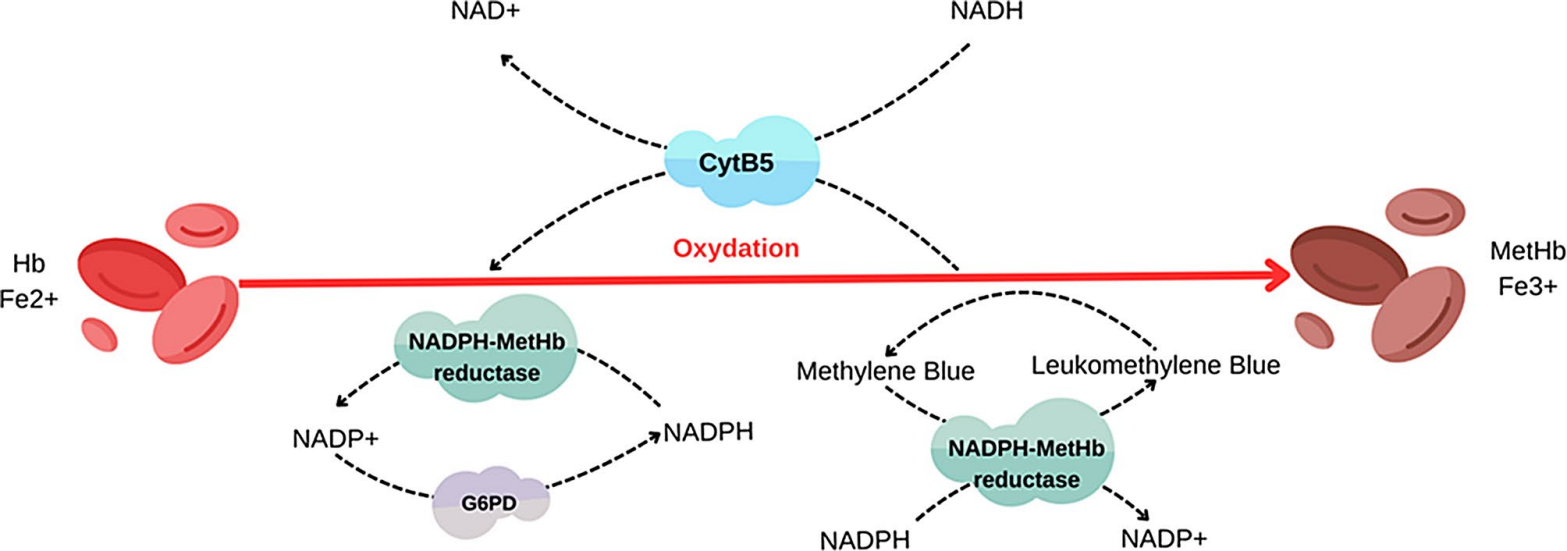
Enzymes involved in methemoglobin (MetHb) homeostasis. The primary process for reducing MetHb is mediated by cytochrome b5-methemoglobin reductase (CytB5), which utilizes the reduced form of nicotinamide adenine dinucleotide (NADH) formed during glycolysis to reduce methemoglobin back to functional hemoglobin. Additionally, a smaller contribution is made by nicotinamide adenine dinucleotide phosphate dependent methemoglobin (NADPH-MetHb) reductase. Under normal physiologic circumstances, NADPH-MetHb reductase contributes very little to the reduction of methemoglobin, but under oxidative stress, the function of this alternative reduction pathway can be enhanced by the presence of exogenous electron shuttle, such as methylene blue, which allows NADPH to reduce methemoglobin

Methemoglobinemia is an uncommon but potentially life-threatening condition caused by the oxidation of the iron in hemoglobin, converting it from the normal ferrous (Fe2+) state to the ferric (Fe3+) state [2]. This altered form of hemoglobin, known as methemoglobin (MetHb), is incapable of effectively transporting and releasing oxygen to the body’s tissues. As a result, the oxygen-hemoglobin dissociation curve shifts to the left, leading to functional anemia and reduced oxygen delivery. Normally, the cytochrome b5 reductase (CytB5) enzyme pathway maintains a low basal level of MetHb, typically around 1.0-1.5% [3]. However, in certain situations, such as during an oxidant challenge, a secondary pathway involving glucose-6-phosphate dehydrogenase (G6PD) and nicotinamide adenine dinucleotide (NAD^+^) can become more prominent in helping to manage elevated MetHb levels (Fig. 1) [4, 5].

Methemoglobinemia can be either inherited or acquired. The inherited, form is attributed to mutations in the CYB5R3 gene, which encodes the CytB5 enzyme. This genetic condition presents in two subtypes: type I, characterized by an unstable enzyme localized to red blood cells, leading to methemoglobin levels exceeding 25% and symptoms such as cyanosis, headache, fatigue, and dyspnea; and type II, caused by variants that diminish enzyme expression or activity across all tissues, resulting in methemoglobin levels ranging from 8 to 40% and severe neurological manifestations. The distinction between type I and II methemoglobinemia stems from the differential expression of CYB5R isoforms in red blood cells compared to other cell types [6]. In addition to the autosomal recessive forms of methemoglobinemia, a rare group of hemoglobin variants known as M group variants (HbM) can arise from autosomal dominant mutations in the genes encoding the globin chains. These HbM variants are characterized by structural abnormalities that lead to heme iron auto-oxidation and cyanosis, often without manifesting other significant symptoms [7]. To date 13 distinct HbM variants have been identified, with some named after the geographic locations in which they were first discovered. HbM variants affecting the alpha-globin chain typically cause cyanosis evident at birth, while those involving the beta-globin chain become apparent later as fetal hemoglobin is replaced by the adult form [8].

Acquired MetHb, on the other hand, is caused by the consumption of certain drugs or exposure to toxins that hasten the oxidation of hemoglobin, resulting in a temporary increase in MetHb levels. Some common agents associated with acquired MetHb include: nitrites, such as those found in certain medications (e.g., benzocaine, dapsone, nitrates), chemicals such as aniline dyes and aromatic compounds, certain antibiotics (e.g., sulfonamides), and ingesting contaminated well water and food containing nitrate [9, 10] (Table 1).

**Table 1:** Substances that can cause MetHb [9, 11]

Inorganic Agents Nitrates—fertilizers, contaminated well water, preservatives, industrial products Chlorates Copper sulfate—fungicides
**Organic Nitrites/Nitrates** Amyl nitrite Isobutyl nitrite Sodium nitrite Nitroglycerin Nitroprusside Nitric oxide Nitrogen dioxide Trinitrotoluene, combustion products
**Drugs** • **Analgesics/antipyretics**: acetaminophen, fentanyl, phenacetin, Phenazopyridine, celecoxib • **Local anesthetics**: benzocaine, lidocaine, prilocaine • **Anticonvulsivants**: phenobarbital, phenytoin, Sodium Valproate • **Anti-Infective-drugs**: •Antimalarials: primaquine, chloroquine •Antimicrobials: sulfonamides, nitrofurans, •Antitubercolosis: P-aminosalicylic acid, Rifampicin, Dapsone • **Antineoplastic agents**: cyclophosphamide, ifosfamide, flutamide • **Others**: Rasburicase, Methylene blue (high dose or in G6PD-deficient patients)

In the presented case, the patient’s history did not indicate any genetic conditions, and baseline MetHb levels were normal. As such, the clinicians focused their investigations on potential foodborne poisoning. Given that tests on the consumed meat were unremarkable, the clinicians inferred that the Swiss chard was the likely source of the oxidative insult. This hypothesis aligns with findings from a recent systematic review on food-induced MetHb, which identified nitrites and nitrates as the primary oxidizing agents implicated [12]. The review further noted that the most common scenarios for food-related methemoglobinemia involve children consuming improperly stored vegetables (30%), accidental ingestions (27%), and errors during meat curing processes (27%) [12].

Nitrates and nitrites play a central role in the development of methemoglobinemia [13]. Nitrates are relatively less harmful than nitrites, but they can be transformed into nitrites through various processes, often due to improper storage or cooking of certain vegetables like spinach, beets, and carrots [14]. Nitrites are commonly used in the food industry to maintain the pinking of meat or in high doses to preserve meat and kill bacteria [15]. A target concentration of nitrate nitrogen for food of less than 100 ppm is desirable for infants, and some commercially prepared infant food vegetables are monitored voluntarily by manufacturers for nitrate content [16]. Children are particularly vulnerable to methemoglobinemia related to nitrites and nitrates. They have lower stomach acid production, leading to a greater presence of nitrate-reducing bacteria in their gut flora. Additionally, the methemoglobin reductase system, which helps manage elevated methemoglobin levels, only fully matures around six months of age. This combination of increased nitrite/nitrate exposure and immature reductase system puts young children at higher risk for developing methemoglobinemia from food sources [12, 17].

### Clinical features and diagnostic approach

To diagnose this disease and distinguish between hereditary and acquired forms, it is crucial to obtain a detailed clinical and family history, assess for consanguinity, and review environmental and drug exposures. For acquired forms, the cyanosis is typically of acute onset, so promptly investigating a history of drug or toxin exposure is important. In contrast, a longstanding family history of cyanosis, dusky-coloured skin, or blue sclera would suggest congenital forms [8].

The clinical manifestations of acute acquired MethHb are contingent on the percentage of methemoglobin saturation. Saturation levels below 10% typically do not cause symptoms, while levels between 10% and 25% result in cyanosis. As MethHb levels increase, individuals may experience symptoms such as headache, fatigue, dizziness nd shortness of breath at levels ranging from 35 to 40% [18]. Levels reaching 60% can lead to arrhythmias, seizures, lethargy, and stupor. When surpassing 70%, it may cause vascular collapse and death [19, 20].

Patients who experience a sudden onset of cyanosis and hypoxia that does not improve despite 100% oxygen therapy, as indicated by arterial blood gas results and the distinct dark red/chocolate brown color of the arterial blood during phlebotomy, may indicate an increase in MetHb [21]. Confirming the diagnosis involves measuring MetHb levels, obtaining positive co-oximetry results, and identifying an oxygen saturation gap greater than 5% between arterial blood gas oxygen saturation and pulse oximeter reading (SpO2-SaO2) [22].

It is crucial to consider the hemoglobin levels of the patient and compute methemoglobin levels using the formula Hemoglobin in grams per deciliter multiplied by MetHb percentage (g/dL x MetHb%). This method will yield a more precise measurement of MetHb level in grams per deciliter, enhancing accuracy when assessing residual functional hemoglobin levels [23]. In our case report the patient had only 3.2 gr/dl of functional hemoglobin left as the Hb level was 11.4 gr/dl, and MetHb saturation was 72.7%. It is noteworthy that despite cyanosis being commonly associated with MetHb, a systematic review has revealed that cyanosis and hypoxemia are not always present in acquired MetHb cases. This underscores the significance of using co-oximetry as a diagnostic tool, which utilizes at least four light wavelengths to measure various forms of hemoglobin including oxyhemoglobin, deoxyhemoglobin, CO-Hb, and Met-Hb [23, 24]. Alternative technologies, such as optoacoustic or spectral sensors, have been proposed to detect dysfunctional hemaglobin types in the bloodstream. Similarly, non-invasive methods based on in silico models have been explored. These alternative options may offer supplementary support for the clinical management of methemoglobinemia going forward [25].

Key diagnostic tests in the evaluation of suspected congenital methemoglobinemia include: assessing MetHb levels, measuring CYB5R enzyme activity, and conducting genetic testing. In individuals with congenital CYB5R3 deficiency, the activity of the CYB5R enzyme is typically reduced to less than 20% of the normal level. Electrophoresis can identify HbM variants caused by mutations in globin genes and DNA sequencing of the CYB5R3 gene can confirm the diagnosis. Emerging next-generation sequencing (NGS) technologies facilitate the detection and characterization of significant genetic variants in patients with rare erythrocyte disorders such as methemoglobinemia, thereby expediting the differential diagnosis [26].

### Therapy

The management of methemoglobinemia in infants and children is guided by multiple considerations, such as symptoms, the overall percentage of methemoglobin, the underlying cause of the methemoglobinemia, and the patient’s age. Asymptomatic individuals with methemoglobin levels below 20% typically do not necessitate any specific intervention beyond the avoidance of oxidizing agents. For acquired methemoglobinemia, treatment is recommended at levels of 20% in symptomatic patients and 30% in asymptomatic patients. Patients with hereditary methemoglobinemia can tolerate higher MetHb levels, with some remaining asymptomatic up to 30–40% [8].

Prompt initiation of therapy involves halting exposure to the triggering oxidant stressor and providing high-flow oxygen. Methylene blue (MB) is the most effective antidote, as it stimulates NADPH MetHb reductase (Fig. 1) [5]. The suggested administration is 1 to 2 mg/kg via intravenous infusion of a 1% solution over a span of 5 min. If required this dosage can be repeated within an hour [27, 28]. It should be pointed out that the administration of MB is controversial in patients with known G6PD deficiency, due to the risk of hemolytic anemia [29, 30]. MB is an oxidizing agent, while its metabolic product, leukomethylene blue, is a reducing agent. Large doses of MB can lead to higher levels of the oxidizing agent itself rather than the reducing metabolite, resulting in hemolysis. Moreover in G6PD deficiency, the lack of sufficient NADPH production prevents the reduction of MB to the less oxidizing leukomethylene blue, consequently, MB therapy may be ineffective in G6PD-deficient patients [5]. That is why in the latest recommendations for diagnosis and treatment of MetHb it is emphasised that patients should be tested for G6PD deficiency before receiving MB treatment, and in case of emergency include, the patient’s family history of G6PD deficiency should be checked before administering MB [8]. In this case options include administering MB in a low dose combined with ascorbic acid, or not using it at all and providing ascorbic acid only [31]. Ascorbic acid antioxidant capacity enables direct reduction of MetHb levels, however the process is slow and often requires multiple doses over 24 h. It is the preferred treatment when MB is unavailable or in cases of MetHb with G6PD deficiency. Dosing in children ranges from 0.5 g every 12 h for 16 doses to 1 g every 4 h for 8 doses. In case of failure of the listed treatments, other treatment options include exchange transfusion and hyperbaric oxygen therapy [4].

In McNulty’s systematic review on food-induced metaemoglobinemia most cases resulted in survival (35 deaths out of 568 cases reported), even when experiencing severe methemoglobin levels reaching up to 89%, as long as MB was promptly administered. None of the fatalities had received MB [12]. There were no deaths reported from cases of favism crisis which, in general, had lower methemoglobin fractions (maximum 15.8%). This emphasizes the significance of promptly identifying the toxidrome and initiating treatment with antidotes.

Due to the infrequency of this condition, the existing literature mainly comprises collections of case studies. A recent study conducted at five Italian pediatric emergency departments between 2007 and 2010 reported nineteen instances of acquired MetHb. The median age was 8.23 months, with a median time of 6 h from trigger to symptom onset. Improper food preservation, particularly vegetable broth, was identified as the primary source of poisoning in most cases. MB treatment was administered to 14 patients (73.7%), all of whom survived [32].

Another similar case series formulated by a Canadian pediatric emergency department involved 10 patients with acquired MetHb. Half of them were affected by hematologic malignancies, so the trigger of poisoning was easily identified as pharmacological (dapsone and rasburicase). The other half presented a previously undiagnosed G6PD deficiency and concomitant explosion to fava beans and topical menthol. Five of the patients were treated with packed red blood cell transfusion, two of them were given MB (MetHb saturation of 19% and 22%), one was treated with ascorbic acid and the last received supportive therapy with fluids and oxygen [33].

This case report highlights the importance of understanding and properly managing MetHb. High-fidelity simulation, as demonstrated by Alagha et al., can be a valuable tool for teaching this topic. Their simulation allowed participants to evaluate and treat MetHb in a safe, controlled setting. It was followed by a debriefing and discussion to review aspects of patient care, including medical knowledge, communication, and practice-based learning. The educational content and effectiveness were evaluated through feedback and a post-simulation survey. The survey showed that after the simulatiothin, 92% of emergency medicine residents felt confident in recognizing and treating MetHb, compared to 62.5% before. Overall, the results indicate that simulation-based training can improve recognition and management of MetHb [34].

In conclusion, acquired MetHb in the pediatric emergency department is an uncommon occurrence, but it should be considered when a patient presents with cyanosis, persistent hypoxia, “cyanosis-saturation gap,” and dark brown blood. Familiarity with this toxidrome and its clinical manifestations, despite its rarity, enables clinicians to initiate timely and suitable antidote therapy which can often be life-saving.

## Data Availability

The datasets used and/or analyzed during the current study are available from the corresponding author on reasonable request.

## Abbreviations

MEtHb: Methemoglobinemia
VBG: Venous blood gas analysis
G6PD: glucose-6-phosphate dehydrogenase
MB: Methylene blue
